# Identification of Novel Malaria Antigens Expressed on the Surface of RBCs Infected with *Plasmodium falciparum*

**DOI:** 10.3390/vaccines14050418

**Published:** 2026-05-06

**Authors:** Ahmad Rushdi Shakri, Alok Das Mohapatra, Jhasketan Badhai, Aditya Anand, Alvin Varghese, Dipak Kumar Raj

**Affiliations:** 1 Department of Internal Medicine, Morsani College of Medicine, 3720 Spectrum Blvd, University of South Florida, Tampa, FL 33612, USA; arshakri@usf.edu (A.R.S.); jhasketanbadhai@usf.edu (J.B.); adityaa2@usf.edu (A.A.); varghesealvin24@gmail.com (A.V.); 2 Department of Pathology and Laboratory Medicine, Warren Alpert Medical School, Brown University, Providence, RI 02912, USA

**Keywords:** malaria, plasmodium, falciparum, vaccine, infected RBC membrane

## Abstract

**Background/Objectives:** Malaria affects almost half of the world’s population and causes more than 600,000 deaths annually. Young children in malaria-endemic areas have the highest mortality rate because of their immature immune systems. Global efforts to control the disease have had limited success, with two WHO-recommended pre-erythrocytic malaria vaccines showing suboptimal efficacy; no vaccine has yet been approved against the blood stages of the parasite that causes the clinical symptoms of malaria. Therefore, there is an urgent need to identify new vaccine candidates against the parasite’s blood stages to achieve protection against the disease. **Methods:** Previous studies in our lab identified a few potential vaccine candidates expressed on the surface of malaria-parasite-infected RBCs using sera from disease-resistant children from malaria-endemic regions and a phage-displayed cDNA library generated from *P. falciparum*. In an innovative approach, we successfully immunized mice using live *Plasmodium falciparum*-infected red blood cells {Pf-iRBCs (L)}, the membrane fraction of *P. falciparum*-infected RBCs {Pf-iRBCs (M)}, and live uninfected human red blood cells (hRBCs) in suitable adjuvants. The polyclonal sera produced against Pf-iRBC immunizations were evaluated for specificity and parasite inhibition in vitro and used in a phage display biopanning assay to identify novel antigens on the surface of Pf-iRBCs. **Results**: Our data indicate that the polyclonal serum produced in BALB/cJ mice, against live Pf-iRBC (L) and their membrane fraction, specifically interacts with surface antigens of parasitic origin on Pf-iRBCs. Additionally, the anti-Pf-iRBC polyclonal serum exhibits significant parasite-killing activity in the in vitro growth inhibition assay (GIA). We have identified both known and novel antigens associated with the Pf-iRBC membrane using phage display cDNA library screening assays. **Conclusions:** As a proof of concept, our phage display screening identified antigens known to be associated with the Pf-iRBC membrane. Additionally, we identified several unknown Pf-iRBC antigens predicted to be associated with Pf-iRBC membrane (PlasmoDB), suggesting that our approach has the potential to identify novel antigens yet to be evaluated as vaccine candidates against falciparum malaria. In our follow-on studies, we will evaluate the newly identified antigen using an integrated in vitro and in vivo challenge experiment. These studies form the core supporting data for further evaluation of the vaccine potential of novel Pf-iRBC antigens and for follow-on vaccine trials in non-human primates, with an ultimate goal of a malaria vaccine for humans.

## 1. Introduction

The life cycle of malaria parasites involves two hosts: the female Anopheles mosquito and the human host. The infection begins when an infected female Anopheles mosquito injects sporozoites into the host during a blood meal. In the pre-erythrocytic stage, the sporozoites travel to the liver and invade hepatocytes. During parasite development within hepatocytes, the parasite multiplies and produces a large number of merozoites, which are released into the bloodstream, initiating the erythrocytic stage. During the erythrocytic phase of development, parasites cycle through four asexual blood stages: the ring, trophozoite, schizont, and merozoite stages. Additionally, a small percentage of these parasites develop into male and female gametocytes during this blood stage. When a mosquito bites an infected person, it ingests male and female gametocytes. Inside the mosquito, these develop into sporozoites, which can then infect healthy humans during subsequent blood meals, spreading the infection.

*P. falciparum* malaria leads to considerable illness and death, mostly in poor and developing countries, in Africa, South America, and Southeast Asia. Worldwide, there were 282 million reported cases of malaria and more than half a million deaths in 2025 [1]. The current situation necessitates the development of highly effective vaccines against malaria, yet the vaccine development pipeline is limited, with most candidates targeting only a few parasite antigens [2,3]. Given the challenges we face, it is essential to explore innovative strategies for identifying new vaccine candidates for falciparum malaria.

Currently, the most advanced malaria vaccine, RTS, S, recommended by the WHO for human use, targets the sporozoite-stage protein, the circumsporozoite protein (CSP). This vaccine induces antibodies that block sporozoite invasion of hepatocytes [4]. Although the possible involvement of cell-mediated immune responses has been sought, these responses have yet to be reliably linked to RTS, S-mediated protection [4]. Critical challenges to targeting CSP-based vaccine include: (1) the short duration of exposure of CSP to circulating vaccine-induced antibodies [5], thus requiring very high concentrations of high-affinity antibodies for vaccine efficacy (>100 EU/mL to prevent 50% of infections [6]) and (2) the lack of expression of CSP on blood-stage parasites, as when a single sporozoite evades vaccine-induced antibodies, an uncontrolled blood-stage infection can follow. In the definitive Phase III trial of RTS, S, these challenges resulted in a vaccine efficacy of 17.3% against severe malaria in infants when given in four doses, which declined to 10.3% when given in three doses—neither outcome was statistically significant and acceptable for human use [7]. Recently, the R21/Matrix-M vaccine, the second malaria vaccine approved by the WHO, showed significant efficacy in reducing the incidence of clinical malaria (characterized by parasitemia with fever) by 75–77%. This promising result comes from a Phase IIb trial conducted in an area with high seasonal transmission of the disease [8,9]. However, the long-term effectiveness of R21/Matrix-M is yet to be evaluated. Additionally, the efficacy of R21/Matrix-M against severe malaria is still unclear [10].

Since parasites that evade pre-erythrocytic vaccine immune responses may lead to severe blood-stage infections, there is a pressing need to develop vaccines targeting the blood stages of the parasite [11]. Despite the pressing need for blood-stage vaccines, the development pipeline is still significantly limited. At present, only three clinical trials for *P. falciparum* blood-stage vaccines are actively recruiting participants, according to clinicaltrials.gov {RH5.2-VLP in Matrix-M (NCT05790889), RH5.1 combined with R21/Matrix-M (NCT06958198), and full-length MSP-1 with GLA-SE adjuvant (NCT07124156)}. Both the approved vaccine candidates are designed to lower parasite replication by activating humoral responses that prevent merozoite invasion of red blood cells. However, previous studies have demonstrated significant antigenic variation in MSP-1 [12,13,14] and, despite strong immunogenicity, this antigen failed to generate protection in Phase IIb studies [15]. Rh5 has limited polymorphism in field isolates [16,17] and is essential for erythrocyte invasion [18], anti-Rh5 can block erythrocyte invasion [19], vaccination with Rh5 protects against *P. falciparum* challenge in non-human primates [20], and vaccination of humans results in a modestly reduced *P. falciparum* replication rate in controlled human malaria infection (CHMI) studies, but only at high anti-Rh5 concentrations [21].

To address the challenges in developing an effective vaccine against the blood stage of malaria, we employed a modified whole-proteome differential screening method, which involved using serum from mice immunized with either live *Plasmodium falciparum*-infected red blood cells (Pf-iRBC, L) or their membrane fractions (Pf-iRBC, M) to identify novel antigens targeted by antibodies on the membranes of infected red blood cells.

## 2. Materials and Methods

### 2.1. Malaria Parasite Culture

*P. falciparum* strains 3D7 and W2 were procured from MR4. In addition, NIH 04122821, a parasite isolate from a child, was collected from our field site in Tanzania and subsequently adapted in the laboratory for in vitro culture at the NIH. The parasites were cultured in vitro according to the methods described earlier by Trager and Jensen [22], with minor modifications. Briefly, the parasites were cultured in growth medium comprising RPMI 1640 medium supplemented with 25 mM HEPES (Cat No. BP310-500, Fisher Bioreagents, Pittsburgh, PA, USA), 5% human O+ erythrocytes, 5% Albumax II (Invitrogen, Carlsbad, CA, USA), 24 mM sodium bicarbonate (Cat No. 25080-094, Gibco, Norristown, PA, USA), and 10 μg/mL gentamycin (Cat No. 15710-064, Gibco, USA) at 37 °C in a controlled atmosphere of 5% CO_2_, 1% O_2_, and 94% N_2_.

### 2.2. Harvest of Mature P. falciparum Parasites

**Figure 1:**
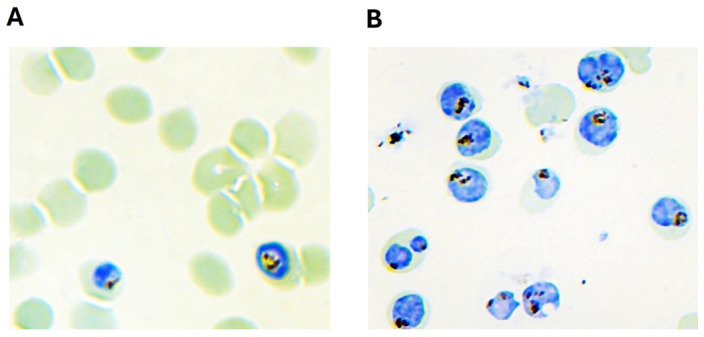
Magnetic enrichment of mature stages of *P. falciparum*-infected red blood cells (Pf-iRBCs): Panel (**A**) and (**B**), representative image shows synchronized in vitro cultures of the 3D7 Pf-iRBCs stained with Giemsa before and after magnetic enrichment. Both panels were observed under a light microscope at 100× magnifications.

Parasites were cultured in fresh human red blood cells (hRBCs), and a high, healthy parasitemia was achieved by replenishing the growth media every 12 h. *P. falciparum* parasites were tightly synchronized through three rounds of sorbitol treatment (Cat No. 194742, MP Biomedicals, Santa Ana, CA, USA), and mature blood-stage parasitized RBCs were further enriched using magnetic column techniques to >90% [23] as shown in Figure 1. Live infected RBCs were collected and used for mice immunization.

### 2.3. Immunization of Mice with Live Pf-iRBC (L)

Female BALB/cJ mice 6–8 weeks of age were immunized with live Pf-iRBCs for polyclonal antibody production. Pre-immune sera were collected by tail bleed from each group (*n* = 5) of mice before immunization. The magnetic column-enriched (>90% parasitemia) *P. falciparum*-infected 10^8^ parasitized RBCs mixed with CpG 1826/TLR-9 liposomes in a final volume of 100 μL (1:1 *v*/*v*) per dose were administered subcutaneously to mice. After administering three doses of immunogen at 3-week intervals, blood was collected from the mice immunized mice by cardiac puncture 21 days after the final injection. The immune sera were harvested from whole blood by centrifugation at 1500 *g* for 10 min and then pooled for further evaluation.

### 2.4. Separation of Pf-iRBC (M) Fraction and Mouse Immunization

Pf-iRBCs were collected as described in Section 2.2, and the membrane fraction was produced by a freeze–thaw cycle using a −80 °C freezer. Lysed RBCs were suspended in chilled sterile 1× PBS, and the pellet was collected by centrifugation at 20,000 *g* for 10 min at 4 °C. The pellet was washed 3–4 times with sterile 1× PBS until no hemoglobin (red color pellet) was observed. The suspended membrane fraction was separated from the dark parasite pellet using a Percoll gradient (30% to 70%) at 20,000 *g* for 10 min [24]. The Pf-iRBC membrane component was formulated at a 1:1 *v*/*v* ratio with TiterMax Gold adjuvant (Cat No. G-20, CytRx Corporation, Los Angeles, CA, USA). Pre-immune sera were collected by tail bleed from each group (n = 5) of mice before immunization. Mice were then subcutaneously immunized three times with 100 μL of the above formulation at 3-week intervals. Blood samples were collected through cardiac puncture from the immunized mice 21 days after the final dose. The immune serum was harvested from the collected blood by centrifugation for further evaluation.

### 2.5. Depletion of Undesired Anti-hRBC Antibodies

**Figure 2:**
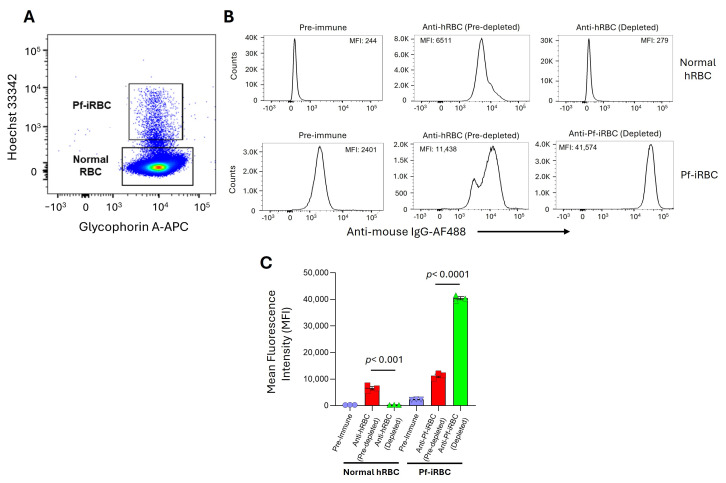
Specific binding of anti-Pf-iRBC (L) to *P. falciparum*-infected red blood cells: (**A**) Representative dot plot showing the gating strategy used to evaluate binding of antibodies in polyclonal mice sera to normal human red blood cells (hRBCs) and *P. falciparum*-infected RBCs (Pf-iRBCs). Normal hRBCs were identified by Glycophorin A^+^/Hoechst 33342^−^ (lower gate), whereas Pf-iRBCs were identified as Glycophorin A^+^/Hoechst 33342^+^ (upper gate). (**B**) Representative histogram plots depicting antibody binding to normal hRBCs and Pf-iRBCs. Post-immune sera were used before or after depletion over normal hRBCs to remove hRBC-specific antibodies. Binding was detected using anti-mouse IgG-AF488, and mean fluorescence intensity (MFI) values are indicated. Enriched sera of Pf-iRBC (L) depleted over hRBC sera showed markedly increased reactivity toward Pf-iRBCs (MFI = 41,574) with minimal binding to normal RBCs (MFI = 279). (**C**) Quantitative analysis of fluorescence intensity demonstrating significant differences between groups (*p* < 0.001), confirming specific recognition of infected erythrocytes by hRBC-depleted immune sera. Depletion of RBC-specific antibodies enhanced the specificity of post-immune serum toward Pf-iRBCs (L) while reducing binding to normal RBCs (*p* < 0.001). The colored shapes in each column of sub-figure (**C**) indicate MFI values obtained from samples analyzed in triplicate. The flow cytometry experiments were repeated three times, and *p*-values were calculated by unpaired *t*-test.

Polyclonal antibodies targeting normal human red blood cells (hRBCs) were depleted from anti-Pf-iRBC (L) (live), and Pf-iRBC (M) (membrane) immune sera through repeated adsorption with normal hRBCs. Briefly, pooled immune sera were incubated with hRBCs at a 50% (*v*/*v*) ratio for 30 min at room temperature. The mixture was then centrifuged at 600× *g* for 3 min, following which the supernatant (serum fraction) was carefully collected and transferred to a fresh tube containing new hRBCs. This depletion step was repeated five to six times to reduce the anti-hRBC antibody titer in the immune serum effectively. All subsequent experiments reported in this manuscript used hRBC-depleted anti-hRBC, anti-Pf-iRBC (L), and anti-Pf-iRBC (M) sera, except the flow cytometry experiment presented in Figure 2C.

### 2.6. Gating Strategy and Identification of Pf-iRBC Surface Antigens in Flow Cytometry

To evaluate the binding ability of anti-Pf-iRBC antibodies to *P. falciparum*-infected RBCs, the anti-hRBC-depleted serum sample from Section 2.5 was incubated with Pf-iRBCs and subsequently probed with fluorochrome (AF488)-conjugated anti-mouse IgG. Pf-iRBCs were separated using a dual staining method involving anti-Glycophorin-A antibodies, which bind to the membrane of human red blood cells (hRBC), and Hoechst 33342, which stains the nucleic acid present only in Pf-iRBC (Figure S1). Mixed-stage infected red blood cells at a parasitemia of 5–10% from a 3D7 strain of *P. falciparum* in vitro culture were incubated with either anti-Pf-iRBC (L) polyclonal sera, anti-hRBC polyclonal sera, or pre-immune sera at a concentration of 10% (*v*/*v*) for 12 h. After incubation, the samples were analyzed by flow cytometry (Figure 2).

### 2.7. Western Blot Using Anti-hRBC and Pf-iRBC Polyclonal Antibodies

*P. falciparum* 3D7 strain parasites were cultured in vitro as described in Section 2.1. Cultures with a parasitemia of ~10% were treated with 0.15% saponin [25] (Cat No. 47036, Sigma-Aldrich, Burlington, MA, USA) in PBS, at pH 7.4, on ice for 10 min. After treatment, the cultures were centrifuged at 3000 *g* for 5 min. The resulting parasite pellets were resuspended and washed with cold PBS, then centrifuged at 3000 *g* for 5 min. The centrifugation and resuspension process was repeated three times. The membrane fraction of Pf-iRBC was separated as described in Section 2.4.

The pelleted membrane fraction was dissolved in an SDS sample-loading buffer with a reducing agent (Bio-Rad, San Francisco, CA, USA) and heated to 95 °C for 10 min. The proteins were then separated using 4–20% gradient SDS-PAGE gels (Bio-Rad), followed by transfer to nitrocellulose membranes and blocking with 5% BSA (Cat No. BP9706, Fisher Bioreagents) in 1× PBS (pH 7.4) with 0.05% Tween 20 (Cat No. J20605-AP, Thermo Scientific, Waltham, MA, USA) for 1 h at 25 °C. The membranes were probed overnight at 4 °C with polyclonal anti-hRBC, anti-Pf-iRBC (L), or anti-Pf-iRBC (M) sera at a 1:1000 dilution in 1× PBS (pH 7.4) containing 0.05% Tween 20. A rabbit polyclonal anti-β-actin antibody, diluted 1:4000, was used to visualize β-actin as a loading control. Following the probing step, the membranes were washed in 1× PBS (pH 7.4) with 0.05% Tween 20. Bound antibodies were detected using either an anti-mouse IgG HRP-conjugated secondary antibody (Cat No. A-4416, Sigma-Aldrich, USA) at a dilution of 1:5000 or an anti-rabbit HRP-conjugated secondary antibody (Cat No. A6015, Sigma-Aldrich, USA). The blots were imaged using the LI-COR Odyssey Imaging System (Lincoln, NE, USA).

### 2.8. Immunofluorescence Assays (IFA)

The IFA microcopy was conducted as described in a published manuscript from our lab with minor modifications. Briefly, thin blood smears of the 3D7 strain parasite cultures were prepared and fixed in cold methanol for 20 min. Blood smears were then probed with anti-Pf-iRBC (L), Pf-iRBC (M), or anti-hRBC sera diluted 1:100, as well as with rabbit anti-PfMSP9 (obtained from MR4) diluted 1:500 in PBS with 5% BSA, at pH 7.4. Slides were incubated with the anti-sera for 1 h at 25 °C, followed by three washes in 1× PBS with 0.05% Tween 20. They were then incubated with goat anti-mouse IgG secondary antibodies conjugated with Alexa Fluor 488 and Alexa Fluor 594 (Molecular Probes, Eugene, Oregon). The blood smears were then incubated with DAPI (Sigma-Aldrich) for 10 min (Sigma-Aldrich) to label parasite nuclei, followed by three washes in 1× PBS with 0.05% Tween 20. Slides were sealed with a cover slip using ProLong Gold anti-fade reagent (Invitrogen). Image acquisition was performed using a confocal microscope (ZEISS LSM 900 Airyscan, Jena, Germany) at 63× magnification [26].

### 2.9. Growth Inhibition Assays (GIAs)

GIA experiments were conducted using anti-Pf-iRBC polyclonal serum generated by immunizing mice with Pf-iRBC (L) in CpG 1826/TLR-9 liposomes or Pf-iRBC (M) formulated with TiterMax Gold adjuvant. The control groups were incubated with serum from uninfected human RBC (hRBC) immunization, pre-immune mouse serum, or with complete medium alone. All sera were used at a final concentration of 10% *v*/*v* in complete media. Briefly, depleted anti-Pf-iRBC sera or depleted hRBC control sera were heat-inactivated at 56 °C for 60 min before being used in growth inhibition assays (GIAs). The GIAs were conducted using the 3D7 and W2 strains of *P. falciparum*. To synchronize the parasites to the ring stage, they were treated with 5% sorbitol for three consecutive replication cycles and then cultured until they reached the ring stage. The parasites were maintained at a parasitemia of 0.5% and a hematocrit of 2%. The samples were incubated with either anti-sera or control sera in a final volume of 100 µL within microtiter wells. After 72 h of incubation in a malaria parasite culture chamber, blood films were prepared from each replicate, stained with Giemsa (Cat No. 48900-1L-F, Sigma-Aldrich, USA), and examined by a blinded microscopist who counted the red blood cells (RBCs) infected with malaria parasites. The results from the replicate wells were averaged, the relationship between the treatment groups and parasitemia outcomes across the replicates was analyzed, and the *p* values were calculated using an unpaired *t*-test.

### 2.10. Pf-cDNA Library and Whole-Proteome Differential Screening

A *P. falciparum* blood-stage cDNA expression library, produced from freshly isolated parasites collected from a child at our Tanzanian field site (NIH) [27,28], was used for our biopanning assay. The whole-proteome differential screening was performed as described in the published article from our laboratory [27]. We coated Immulon 4HB (Cat No. 6404, Thermo Electron Corporation, Waltham, MA, USA)) ELISA wells with 100 μL of a 1:100 dilution of depleted Pf-iRBC (L/M) sera for one hour at RT. The coated wells were then washed 5 times with 1× TBST (10 mM Tris-HCl, 150 mM NaCl, 0.05% Tween 20, pH 7.4). Wells were blocked with 2% BSA in 1× TBST for 1 h at RT, followed by the addition of 100 μL of a 1× TBST solution containing 10^7^ phages. Wells were incubated for 1 h at RT for binding of T7-Phage display c-DNA clones to coated anti-Pf-iRBC polyclonal antibodies. The unbound phages were removed by washing wells five times with 1× TBST. Bound phages were then eluted using 100 µL of 5 M NaCl. The eluted phages were then amplified and titered with BLT5403 bacteria according to the manufacturer’s instructions. The amplified phages served as the input for additional rounds of amplification. After three rounds of positive selection, the eluted phages were titered and diluted to 10^5^ phages/mL in 1× TBST buffer. For negative selection, Immulon 4HB ELISA wells were coated with 100 µL of a 1:100 dilution of polyclonal anti-hRBC serum at room temperature. An amount of 100 µL of the diluted phage solution containing 10,000 phages was added to each well and incubated for 1 h at RT. Unbound phages were collected and transferred to another well coated with anti-hRBC polyclonal mouse serum, incubated for an additional 1 h at room temperature before collection. This process was repeated 5 times to remove any non-specifically bound phages during the biopanning assay.

### 2.11. Identification of Novel Antigens Expressed on the Surface of P. falciparum-Infected RBCs (Pf-iRBC)

After the negative selection described in Section 2.10, the phages were titered, and 100 individual plaques were isolated for sequence analysis. The cDNA inserts from these plaques were amplified using PCR using vector-specific primers: T7Select UP (5′-GGAGCTGTCGTATTCCAGTC-3′) and T7Select Down (5′-AACCCCTCAAGACCCGTTTA-3′). The resulting PCR products were then sequenced (using Eurofins Genomics, Louisville, KY, USA). To analyze the sequences of the c-DNA fragments and corresponding genes, we used PlasmoDB’s release 66 version of the software based on the NCBI BLAST algorithm (www.PlasmoDB.org) to identify the best matches, as summarized in Tables S1 and S2.

## 3. Results and Discussion

### 3.1. Magnetic Enrichment of Mature Stage Pf-iRBCs

Using the modified culture method described in the material and method section, we were able to achieve between 10 and 15% healthy initial parasitemia (Figure 1A). Subsequent magnetic enrichment successfully increased the proportion of live Pf-iRBCs to approximately 90% (Figure 1B) and was useful for mice immunization and polyclonal antibody production.

### 3.2. Evaluation of Anti-Pf-iRBC Antibodies for Specificity Using Flow Cytometry

We used antibodies against an antigen (Glycophorin A), primarily expressed on the surface of human RBCs (hRBC), for the gating strategy, and another polyclonal antibody, anti-PfGARP, of parasite origin, expressed on the surface of Pf-iRBCs, for evaluation of our strategy for detection of Pf-iRBC surface antigens [27]. The binding of polyclonal anti-PfGARP antibodies to the Pf-iRBCs in the upper gate and uninfected RBCs in the bottom gate is shown (Figure S1A,B). The data reveal that Pf-GARP antibodies bind specifically to the surface of Pf-iRBCs in the upper gate, with mature parasites and higher nucleic acid content. In contrast, the anti-PfGARP antibodies show little binding to the normal hRBCs in the lower gate (Figure S1A,B). Using the above gating strategy, flow cytometric evaluation of anti-PfGARP indicates a specific interaction between anti-PfGARP and the GARP antigen on the Pf-iRBC surface (Figure S1B). We use a similar gating strategy to evaluate anti-Pf-iRBC for the identification of novel antigens (Figure 2A,B). These observations indicate that (1) our method significantly depleted antibodies against hRBC proteins from anti-Pf-iRBC polyclonal serum (Figure 2C), with a mean fluorescent intensity (MFI) of 279, equivalent to the pre-immune sera, and a similar pattern was also observed by comparing the pre-depleted anti-Pf-iRBC to post-depleted Pf-iRBCs, and in both the cases, the *p* value was highly significant (*p* < 0.001). (2) The depleted anti-Pf-iRBC polyclonal serum contains antibodies specific to the Pf-iRBC surface protein of parasite origin with an MFI 4-fold higher in the post-depleted vs. pre-depleted sera and more than 17-fold higher than the background produced by the pre-immune sera (Figure 2B).

### 3.3. Evaluation of Anti-Pf-iRBC Antibodies in Western Blot

**Figure 3:**
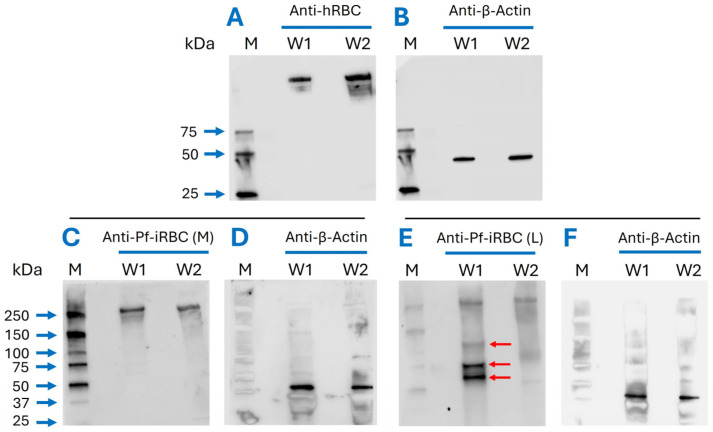
Specific interaction of anti-Pf-iRBC (L) with the protein of parasite origin in Pf-iRBC extract: Western blot using depleted anti-Pf-iRBC (L) polyclonal antibodies demonstrated specific recognition with Pf-iRBC membrane extract (**E**), W1. Lysates from Pf-iRBCs (W1) and hRB (W2) membrane extracts for all blots were separated on 4–20% SDS-PAGE gels and transferred to a nitrocellulose membrane. The membranes were incubated with anti-hRBC (**A**), anti-Pf-iRBC against the membrane (**C**), and anti-Pf-iRBC against live cells (**E**). A polyclonal rabbit anti-β-actin antibody was used to detect β-actin as a loading control (**B**,**D**,**F**). The Western blots are representative of three independent experiments. Densitometry analysis of all blots is presented in Supplementary Figure S5.

We demonstrated that antibodies generated against the Pf-iRBC (L) can specifically recognize multiple antigens of *P. falciparum* origin when probed with the membrane extract of Pf-iRBCs (Figure 3E, W1). In contrast, the depleted anti-hRBC sera show poor binding in the Western blot (Figure 3A) and does not non-specifically bind to any parasite-specific antigens in Pf-iRBC membrane extracts. The Pf-iRBC (M) sera, however, show very weak interactions with antigens from the Pf-iRBC membrane extract (Figure 3C, W1), whereas anti-hRBC sera do not demonstrate any specific interaction. The loading control (β-actin) indicates equivalent loading of membrane extract across all blots (Figure 3B,D,F).

### 3.4. Stage-Specific Immunolocalization of Anti-Pf-iRBC

**Figure 4:**
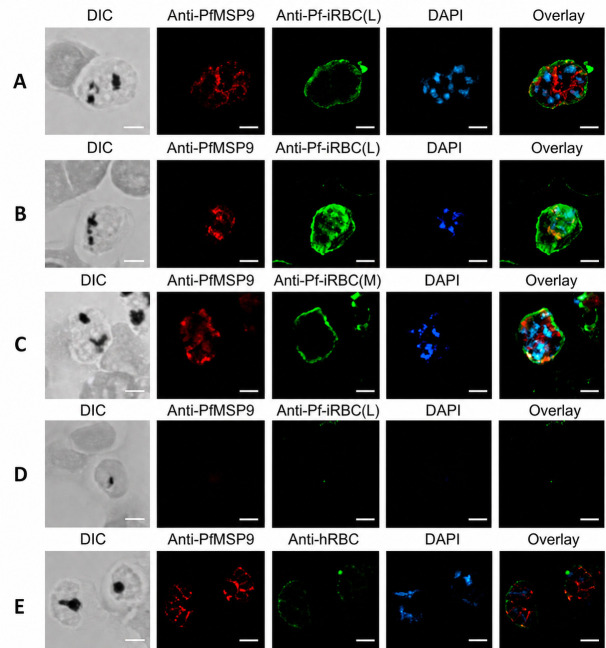
Localization studies with anti-Pf-MSP9 sera indicate that the anti-Pf-iRBC (L and M) did not bind well to the parasite but rather interacted with the surface of mature parasite-infected RBCs. (**A**–**C**,**E**), mature parasite-infected, or (**D**), uninfected hRBCs were probed with rabbit anti-PfMSP9, and counterstained with (**A**,**B**,**D**), anti-Pf-iRBC (L), (**C**), anti-Pf-iRBC (M), and (**E**), anti-hRBC. (**A**,**B**) represent images from independent immunofluorescence assays using the same antibodies and conditions. The nucleic acid of all infected RBCs was stained with DAPI to label parasite DNA. Anti-Pf-iRBC polyclonal sera bound to the mature parasite-infected RBCs but did not co-localize with anti-PfMSP9 binding on the parasite membrane. (**D**,**E**), neither the uninfected RBCs probed with the anti-Pf-iRBC (L), nor the mature-stage-infected RBCs probed with anti-hRBC showed any specific binding. Scale bars represent 2 μm. Images are representative of three independent experiments and the binding pattens are consistent with the mature *P. falciparum* infected hRBC.

We used anti-Pf-iRBC antibodies to immunolocalize surface proteins of parasite origin on Pf-iRBCs by confocal immunofluorescence microscopy. The anti-Pf-iRBC polyclonal antibodies specifically recognized parasite antigens expressed on the membrane of parasitized RBCs with mature trophozoite or schizonts (Figure 4A–C). However, they failed to bind to the surface of normal, uninfected RBCs (Figure 4D), along with merozoite or the ring-stage parasites ). The Pf-iRBC polyclonal sera (L/M) do not bind directly to the parasites or co-localize with anti-PfMSP9 but rather show a pattern similar to the Pf-iRBC membrane in the DIC image (Figure 4A–C). On the other hand, the anti-hRBC polyclonal sera, depleted of hRBC-specific antibodies as mentioned before, failed to bind the membrane of mature parasite-infected RBCs (Figure 4E), suggesting that the protocol used to deplete immune sera of hRBC-specific antibodies was highly effective and supports our flow cytometry data (Figure 2C). An equivalent IFA corresponding to panel D was performed using anti-Pf-iRBC (M). The resulting data showed no detectable fluorescent staining on the RBC membrane, similar to the signal observed with anti-Pf-iRBC (L). Additionally, the uninfected RBCs present in panel C represent the same experimental condition with anti-iRBC (M) and exhibited no fluorescent staining. Therefore, these panels were not included.

### 3.5. Evaluation of Polyclonal Anti-Pf-iRBC Sera in in Vitro GIA

The anti-Pf-iRBC (L) polyclonal sera generated by mouse immunization and hRBC-depletion inhibited parasite growth by 65–70% across two parasite strains compared with controls (*p* < 0.001).

**Figure 5:**
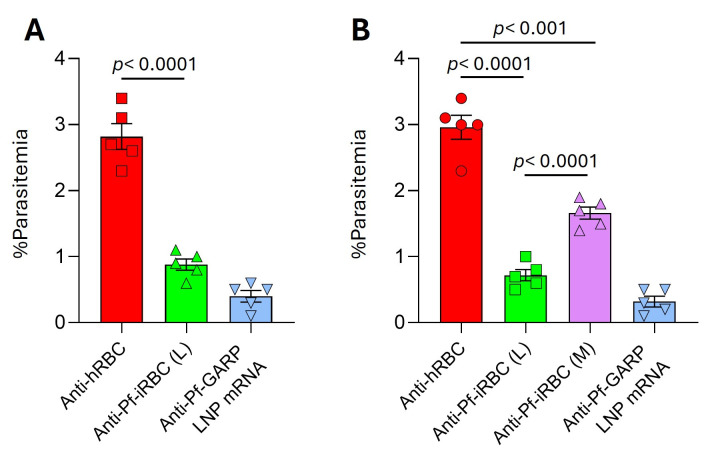
(**A**) Anti-Pf-iRBC (L) shows robust growth inhibition compared to anti-hRBC sera (*p* < 0.0001): GIA was performed on 3D7 using polyclonal anti-Pf-iRBC (L) sera. Ring-stage malaria parasites from 3D7 parasites were cultured in the presence of anti-Pf-iRBC (L) mouse serum at a 1:10 dilution. Anti-hRBC serum was used as a negative control and anti-Pf-GARP LNP mRNA serum as a positive control. Parasites were cultured for 48 h at 37 °C, and ring-stage and early-trophozoite-stage parasites were examined under microscopy. (**B**) Anti-Pf-iRBC (M) sera also show significant growth-inhibition activity in vitro GIA. Polyclonal anti-Pf-iRBC (M) sera inhibited parasite growth by ~45% when compared to the anti-hRBC polyclonal sera (*p* < 0.001). Data are means of three biologically independent replicates. *p* values were calculated using an unpaired *t*-test. Data are representative of three independent experiments.

We consistently observed significant inhibition of parasite growth by anti-Pf-iRBC (L) (*p* < 0.001) in the GIA compared to anti-hRBC sera (Figure 5A,B and Figure S4). We used polyclonal sera produced on an LNP-mRNA vaccine platform [27] as a positive control to validate the experiment. We used a heterologous parasite strain, W2, to evaluate the growth-inhibitory activity of anti-Pf-iRBC antibodies, given its polymorphic nature and higher growth rates than the commonly used laboratory strain, 3D7. The GIA demonstrates significant growth-inhibition activity of ~60% [Pf-iRBC (L) *p* < 0.001] and ~45% [Pf-iRBC (M), *p* < 0.001] in GIA, as illustrated in Figure S4. Furthermore, the anti-Pf-iRBC polyclonal sera treatment reduced parasite growth by 60–65% (all *p* < 0.001) in two parasite lines that were freshly isolated from Tanzanian children. The anti-Pf-iRBC (L and M) growth-inhibition activity on the W2 strain of the parasite, as well as that of the field-isolated strain, appears to be slightly reduced, likely due to the high growth rate of the parasite. Additionally, we are currently using only 10% anti-Pf-iRBC (L and M) serum in the growth inhibition assay (GIA). However, in an in vivo setting, the anti-Pf-iRBC inhibitory activity against specific antigen could provide strong support for further vaccine development. In our GIA experiments, we demonstrated that live anti-Pf-iRBC (L) polyclonal sera exhibited greater growth-inhibition activity than the Pf-iRBC (M) sera (Figure 5B and Figure S4). Flow cytometry, Western blotting and confocal microscopy also demonstrated that the anti-Pf-iRBC membrane fraction produced lower levels of specific antibodies than live parasitized RBCs (Pf-iRBC (L). We used the TiterMax adjuvant with the Pf-iRBC membrane fraction for immunization of mice, as Pf-iRBC (L) with CpG 1826/TLR-9 Liposome adjuvant failed to elicit specific antibodies against Pf-iRBCs in ELISA.

### 3.6. Identification of Novel Antigens for Malaria Vaccine Development

We interrogated the *P. falciparum* blood-stage proteome and identified a few parasite proteins expressed on the surface of Pf-iRBC, which are probable targets of antibody responses to inhibit parasite growth in GIA. We performed differential screening experiments on *P. falciparum* blood-stage phage display cDNA libraries. We screened libraries prepared from minimally culture-adapted parasites to identify most of the proteins expressed on the Pf-iRBC surface, comparable to those expressed by natural strains of the parasite responsible for human malaria in the field. We differentially biopanned 10^7^ clones and continued enriching them in each round of biopanning up to round 3 (Figures S2 and S3) and identified several gene fragments encoding *P. falciparum* antigens with varying degrees of enrichment (Tables S1 and S2). In addition to several known Pf-iRBC antigens and several hypothetical antigens, we identified various housekeeping proteins (Tables S1 and S2) due to their high abundance in the *P. falciparum* phage display cDNA library. We found that using depleted anti-Pf-iRBC (L) polyclonal sera significantly improved identifications of novel antigens compared to depleted Pf-iRBC (M) in the biopanning assay. This was demonstrated by greater enrichment, higher diversity, and enhanced efficiency in identifying known surface antigens of Pf-iRBC in Tables S1 vs. S2. Additionally, we identified novel antigens (Tables S1 and S2) with unknown functions warranting further investigation for their potential as vaccines against *P. falciparum* malaria.

## 4. Conclusions

Malaria remains a leading cause of childhood mortality, and vaccines are urgently needed to attenuate this public health threat. We have used our vaccine antigen discovery method to identify novel vaccine candidates recognized by antibodies expressed on *P. falciparum*-infected human RBCs. Using our optimized phage-display-based approach, we have identified several known antigens that localize to the Pf-iRBC membrane and novel antigens that can be further evaluated as blood-stage vaccines targeting parasite-origin RBC membrane antigens. Interestingly, live cell immunization with Pf-iRBC showed greater immunogenicity compared to the membrane fraction, particularly in terms of functional antibody production. In this manuscript, we show that hRBC-depleted anti-Pf-iRBC polyclonal sera inhibit *P. falciparum* growth up to ~65–70% {Pf-iRBC (L), *p* < 0.0001)} compared to depleted anti-hRBC polyclonal sera in the growth inhibition assay (GIA). This finding is unprecedented, especially given that live cells {(Pf-iRBC (L)} have been used for immunization, and their survival in the bloodstream of mice is limited due to the faster elimination of Pf-infected human RBC by the mice’s immune system.

Additionally, the anti-Pf-iRBC antibodies used in the GIA were further diluted due to serial depletion over hRBC, likely reducing the anti-Pf-iRBC-mediated inhibition of parasite growth in the GIA. However, our observation is consistent with earlier findings that live Pf-iRBC vaccination against falciparum malaria can generate an antibody response and protection [29]. In an important prior study, Pf-iRBC-based protection was observed in Aotus monkeys infected with the human malaria parasite, *P. falciparum* [30]. More recently, multiple low doses of *P. falciparum*-infected red blood cells (Pf-iRBC) were administered to malaria-naïve individuals, with each infection terminated prior to patency, and anti-malaria treatment provided protection to three out of four volunteers [31], supporting our finding. However, immunization with *P. falciparum*-infected membrane fractions, likely rich in antigens such as PfEMP1, apical membrane antigen-1 (AMA-1), glutamic acid-rich protein, and other unknown antigens, has never been evaluated or reported for malaria protection, likely due to its poor functional antibody production and not detectable by standard assays such as Western blot and flow cytometry.

The current malaria vaccine landscape is limited, with only two approved, modestly effective pre-erythrocytic vaccines (RTS, S and R21/Matrix-M). Given the current scenario of vaccine development, a blood-stage vaccine candidate is of utmost importance to prevent or at least reduce the severity of the disease. Because pre-erythrocytic vaccines do not confer sterile protection, there is an urgent need to synergize them with the blood-stage vaccine to prevent the rapid multiplication of parasites that escape the sporozoite vaccines. Our data indicate that polyclonal sera raised against Pf-iRBCs can significantly reduce parasite growth in vitro, suggesting that parasite antigens are present on *P. falciparum*-infected red blood cells. Using our phage display biopanning method, we have identified parasite proteins expressed on infected RBCs, such as *P. falciparum* Knob-Associated Histidine-Rich Protein (Pf-KAHRP) [32] and *P. falciparum* Erythrocyte Membrane Protein 1 (PfEMP1) [33,34], demonstrating the success of our methodology in identifying specific antigens associated with Pf-iRBC and highlighting the reliability of our approach for identifying novel vaccine candidates against falciparum malaria.

We have also identified several novel *P. falciparum* proteins with unknown functions that were predicted by in silico analysis and proteomics data and are more likely to be present on the surface of Pf-iRBC. These include the PF3D7_0108500, PF3D7_1239800, PF3D7_1248700, PF3D7_1361200 and PF3D7_1423700 genes. We are now focusing on antigens that are enriched in our biopanning assays and predicted by in silico analysis to be accessible to parasite-inhibitory antibodies for further evaluation using mRNA and recombinant protein platforms. The assessment will include both in vitro studies and experiments using murine models of malaria. The data generated will provide essential support for advancing the novel malaria antigens identified through our approach. This will further facilitate analysis in primate models and, eventually, in human trials.

## Data Availability

The original contributions presented in this study are included in the article/Supplementary Material. Further inquiries can be directed to the corresponding author.

## Supplementary Materials

The following supporting information can be downloaded at https://www.mdpi.com/article/10.3390/vaccines14050418/s1. Figure S1. Standardization of the gating strategy for flow cytometry assays. Figure S2. Enrichment of phage clones in Rounds 1 to 3 of biopanning process using anti-Pf-iRBC (L) sera. Figure S3. Enrichment of phage clones in Rounds 1 to 3 of biopanning process using anti-Pf-iRBC (M) sera. Figure S4. Growth inhibition assay using *P. falciparum* strain W2. Figure S5: Uncropped images of Western blots (A to F) and densitometry analysis of major protein bands detected in each blot. Table S1. *P. falciparum* genes identified: PfiRBC (L). Table S2. *P. falciparum* genes identified: PfiRBC (M).

## Abbreviations

The following abbreviations are used in this manuscript:

WHO	World Health Organization
RBC	Red blood bell
Pf-iRBC (L)	*Plasmodium falciparum*-infected RBC live
Pf-iRBC (M)	*Plasmodium falciparum*-infected RBC membrane fraction
hRBC	Uninfected human RBC
Rh5	Reticulocyte-binding protein homologue-5
CSP	Circumsporozoite protein
IV	Intravenous
MSP-1	Merozoite surface protein-1
PCV	Packed cell volume
Pf-GARP	*P. falciparum* glutamic acid rich protein
