# Supplementary material for: Identification of Novel Malaria Antigens Expressed on the Surface of RBCs Infected with Plasmodium falciparum
Source: Vaccines (Basel). 2026 May 6;14(5):418. doi: 10.3390/vaccines14050418 (PMC13211579; doi:10.3390/vaccines14050418)
Supplement: Supplementary file 1 [file vaccines-14-00418-s001.zip › vaccines-4252843-supplementary.pdf]

vaccines

Identification of Novel Malaria Antigens Expressed on the Surface of RBCs Infected with *Plasmodium falciparum*

Ahmad Rushdi Shakri [1], Alok Das Mohapatra [2], Jhasketan Badhai [1], Aditya Anand [1], Alvin Varghese [1] and Dipak Kumar Raj [1,*]

Supplementary Figures and Tables

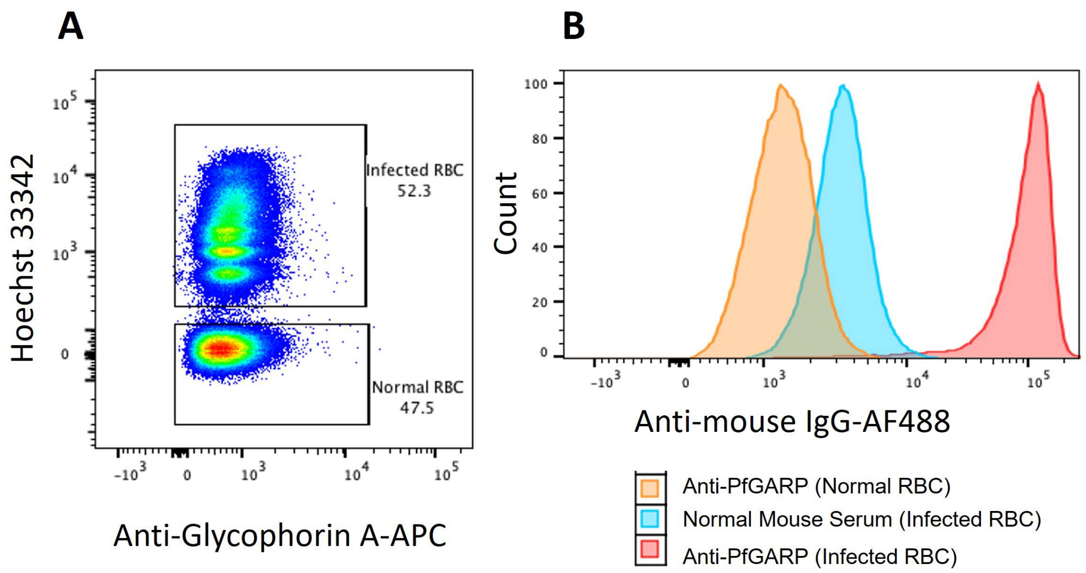

Figure S1. *Standardization of the gating strategy for flow cytometry assays.* A known PfiRBC membrane protein of parasite origin was evaluated in flow cytometry to standardize the gating strategy to ensure specificity of Anti-Pf-iRBC antibodies. Ring stage *P. falciparum* 3D7 parasites were synchronized and cultured to trophozoite stage. The samples were fixed by 2% paraformaldehyde in 1x PBS for 30 minutes. **A,** Normal and Infected RBCs were identified and gated by using Hoechst 33342 and APC-conjugated anti-human Glycophorin A antibody. **B,** Expression of PfGARP on the surface of fixed but not permeabilized infected RBC was determined by flow cytometry, using anti-PfGARP (LNP-m-RNA) as the primary and anti-mouse IgG–FITC as the secondary antibody as shown above. BD FACS Diva 6.1.1 and FlowJo vX.0.7 were used for data collection and analysis respectively.

 https://doi.org/10.3390/vaccines14050418

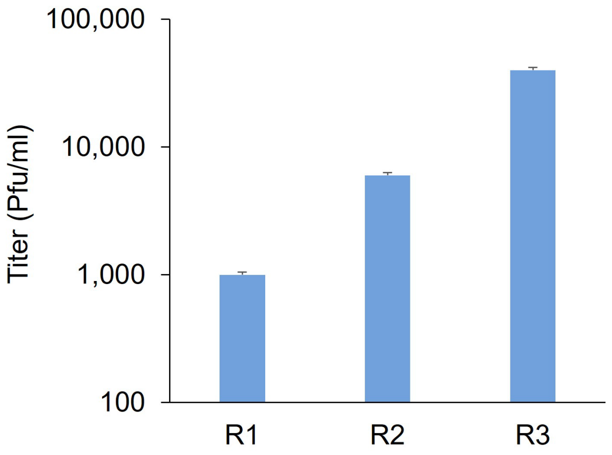

Figure S2. *Enrichment of phage clones in Rounds 1 to 3 of biopanning process using anti-Pf-iRBC (L) sera.* Significant enrichment of the clones for phage containing *P. falciparum* cDNA fragments were observed until third round of biopanning. Enrichment of phages after each round of biopanning of phage display c-DNA library on mice anti-Pf-iRBC (L) antibodies are presented.

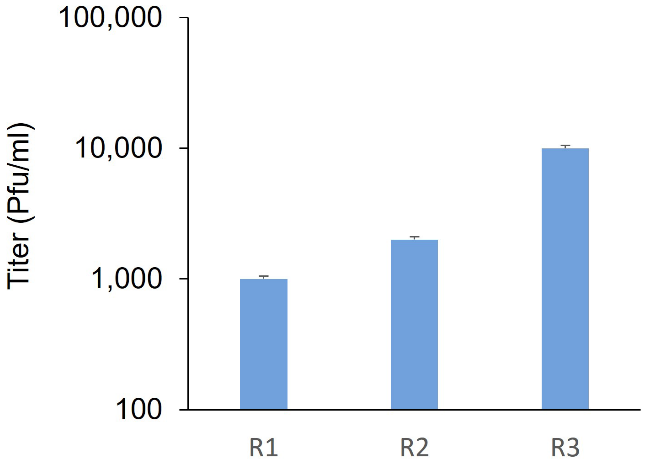

Figure S3. *Enrichment of phage clones in Rounds 1 to 3 of biopanning process using anti-Pf-iRBC (M) sera.* Significant enrichment of the clones for phage containing *P. falciparum* cDNA fragments were observed until third round of biopanning. Enrichment of phages after each round of biopanning of phage display c-DNA library on mice anti-Pf-iRBC (M) antibodies are presented.

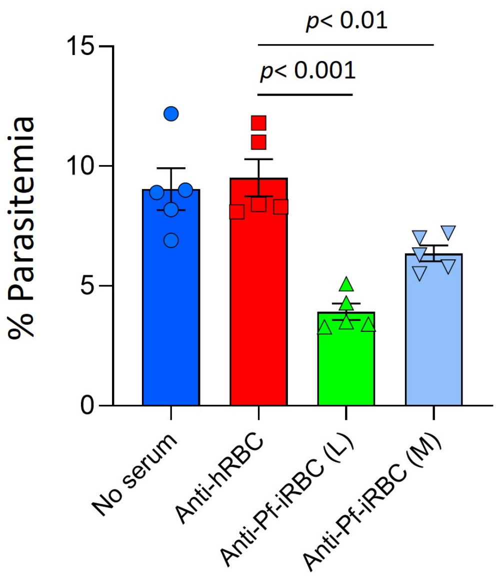

Figure S4. *Growth inhibition assay using P. falciparum strain W2.* Anti-Pf-iRBC shows comparative growth inhibition in a heterologous strain of *P. falciparum*. GIAs performed on W2 strains of the parasite using hRBC depleted polyclonal anti-Pf-iRBC (L) sera generated by immunizing mice with live mature blood-stage parasite-infected RBCs in CpG 1826/TLR9 Liposome adjuvant, or anti-Pf-iRBC (M) polyclonal sera generated by immunizing mice with membrane fraction of mature blood-stage parasites formulated with TiterMax Gold adjuvant. Ring-stage malaria parasites from W2 strain were cultured in the presence of anti-Pf-iRBC (L or M). Mice sera were used at a 1:10 dilution. Negative control was taken as No serum (Blue) and anti-hRBC (Red). Parasites were cultured and parasitemia evaluated as described for Figure 5. Data are mean of three experimental replicates. P values were calculated by unpaired t-test. Data are representative of three independent experiments.

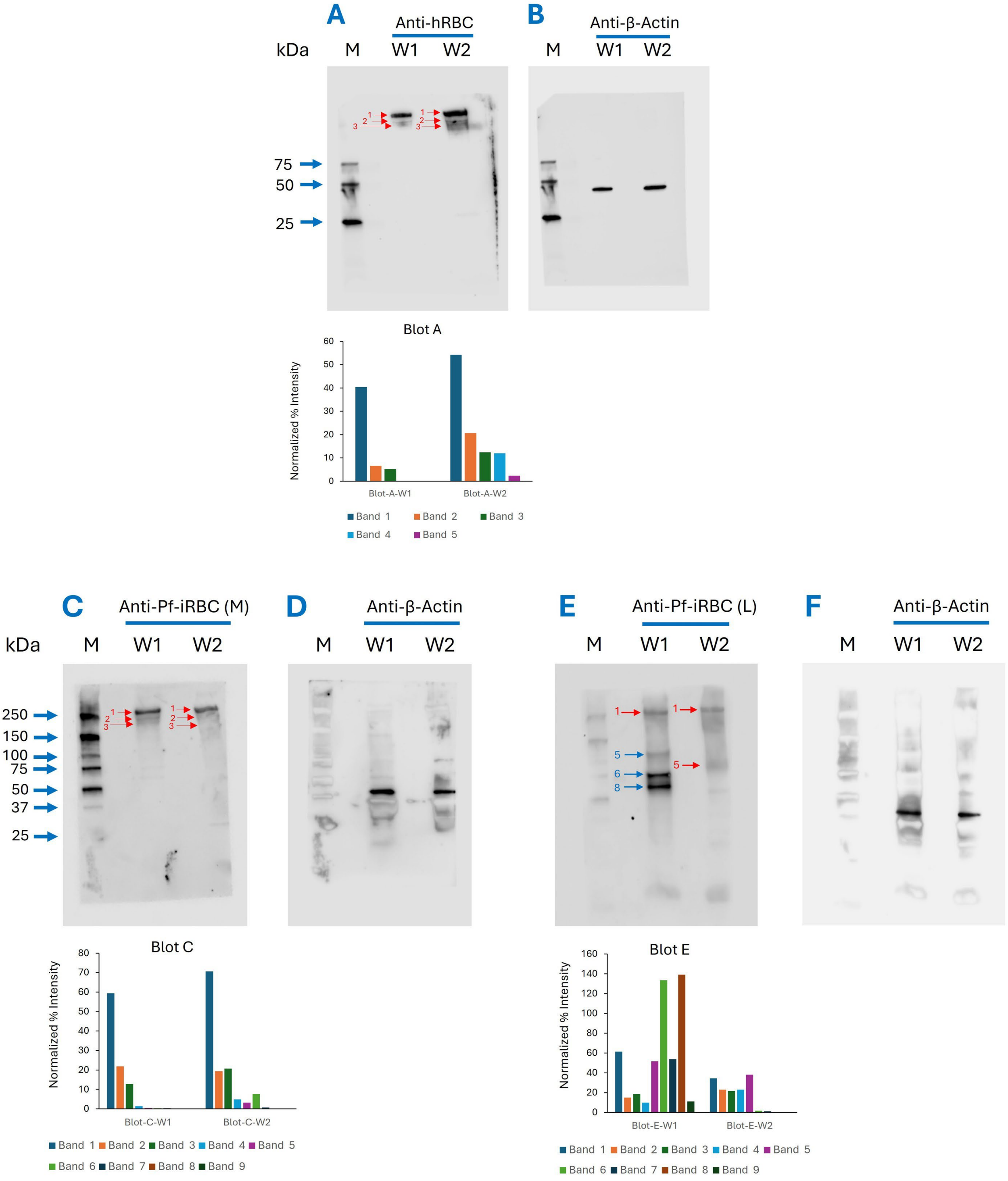

Figure S5: *Uncropped images of Western blots (A to F) and densitometry analysis of major protein bands detected in each blot.* After probing and visualizing the blots using immune sera from various conditions, the blots were stripped and re-probed with rabbit anti-β-actin to detect β-actin as a loading control. Densitometry of protein bands was performed by ImageJ software. Normalized percent intensities of major protein bands detected by the software were determined by dividing the intensity value of the band of interest by intensity value of β-actin band (of the corresponding blot) x 100. Major protein bands are numbered and normalized percentage (%) intensity values are shown in the corresponding bar graphs of each lane. Major protein bands specifically detected by anti-Pf-iRBC (L) sera are indicated by blue markings for bands 5, 6, and 8 in Blot E, Lane W1.

Table S1. *P. falciparum* genes identified: PfiRBC (L).

Gene name	Gene ID	% of Clones
P. falciparum SD17 gene for knob-associated histidine-rich protein	PF3D7_0202000	3
P. falciparum isolate 1971-4 erythrocyte membrane protein 1	PfGN01_060006100	3
P. falciparum SB794136 sera5 gene for serine repeat antigen 5	PF3D7_0207600	3
P. falciparum membrane-associated calcium-binding protein (Pfs40) gene	PF3D7_1108600	1
P. falciparum (isolate V1) S-antigen gene	PfDd2_100040400	32
P. falciparum 3D7 conserved protein, unknown function	PF3D7_0108500	8
P. falciparum 3D7 pantothenate kinase 1	PF3D7_1420600	3
P. falciparum 3D7 conserved Plasmodium protein, unknown function	PF3D7_1239800	1
P. falciparum 3D7 conserved Plasmodium protein, unknown function	PF3D7_1248700	3
P. falciparum 3D7 cytoskeleton associated protein, putative	PF3D7_0708000	3
P. falciparum 3D7 eukaryotic translation initiation factor 3 subunit A, putative	PF3D7_1212700	7
P.falciparum 3D7 nucleic acid binding protein, putative	PF3D7_0202600.1	3
P. falciparum RNA polymerase III largest subunit gene	PF3D7_1329000	1
P. falciparum 3D7 nucleolar protein Nop52, putative	PF3D7_0931100	4
P. falciparum 3D7 DNA/RNA-binding protein Alba 3	PF3D7_1006200	6
P. falciparum 3D7 bromodomain protein 1	PF3D7_1033700	10
P. falciparum 3D7 DnaJ protein/HSPA8-interacting J	PF3D7_1401100	4
P. falciparum isolate SF2 18S ribosomal RNA gene	PfTG01_050036600	3
P. falciparum 3D7 28S ribosomal RNA	PF3D7_0726000	1
	Total	100

P. falciparum genes identified by biopanning of phage display cDNA expression library generated from blood-stage parasites with immune sera generated upon immunization of mice with *P. falciparum*-infected live human red blood cell [Pf-iRBC (L)]. The percentage of clones is representative of relative enrichment of phage clones expressing various parasite-derived proteins. Underlined, *P. falciparum* proteins are known to express on the surface of parasite-infected RBCs.

https://doi.org/10.3390/vaccines14050418

Table S2. *P. falciparum* genes identified: PfiRBC (M).

Gene name	Gene ID	% of Clones
Plasmodium falciparum 3D7 conserved Plasmodium protein, unknown function,	PF3D7_1361200	51
Plasmodium falciparum 3D7 endoplasmin, putative	PF3D7_1222300	12
Plasmodium falciparum 3D7 ribonucleoprotein-associated protein B, putative	PF3D7_1414800	2
Plasmodium falciparum 3D7 knob associated heat shock protein 40	PF3D7_0201800	7
Plasmodium falciparum 3D7 60S ribosomal protein L11a, putative	PF3D7_0719600	3
Plasmodium falciparum 3D7 stearoyl-CoA desaturase	PF3D7_0511200	2
Plasmodium falciparum 3D7 periodic tryptophan protein 1, putative	PF3D7_1237600	11
Plasmodium falciparum 3D7 serine/threonine protein kinase, FIKK family	PF3D7_1039000	4
Plasmodium falciparum 3D7 LMBR1 domain-containing protein, putative	PF3D7_1338000	2
Plasmodium falciparum 3D7 MYND-type zinc finger protein, putative	PF3D7_0602200	1
Plasmodium falciparum 3D7 conserved Plasmodium protein, unknown function	PF3D7_1423700	2
Plasmodium falciparum 3D7 60S ribosomal protein L4	PF3D7_0507100	1
Plasmodium falciparum 3D7 18S ribosomal RNA	PF3D7_0725600	1
Plasmodium falciparum 3D7 heat shock protein 70	PF3D7_0818900	2
Plasmodium falciparum 3D7 conserved Plasmodium protein, unknown function	PF3D7_1308400	1
	Total	100

P. falciparum genes identified by biopanning of phage display cDNA expression library generated from blood-stage parasites with immune sera generated upon immunization of mice with membranes isolated from *P. falciparum*-infected human RBCs. The percentage of clones is representative of relative enrichment of phage clones expressing various *P. falciparum* parasite-derived proteins. Underlined *P. falciparum* proteins are known to express on the surface of parasite-infected RBCs or aid in remodeling of host erythrocytes.
